# Identification of heart failure subtypes using transformer-based deep learning modelling: a population-based study of 379,108 individuals

**DOI:** 10.1016/j.ebiom.2025.105657

**Published:** 2025-03-19

**Authors:** Zhengxian Fan, Mohammad Mamouei, Yikuan Li, Shishir Rao, Kazem Rahimi

**Affiliations:** a Deep Medicine, Nuffield Department of Women’s & Reproductive Health, University of Oxford, Oxford, United Kingdom

**Keywords:** Heart failure, Machine learning, Deep learning, Disease subtyping, Clustering

## Abstract

**Background:**

Heart failure (HF) is a complex syndrome with varied presentations and progression patterns. Traditional classification systems based on left ventricular ejection fraction (LVEF) have limitations in capturing the heterogeneity of HF. We aimed to explore the application of deep learning, specifically a Transformer-based approach, to analyse electronic health records (EHR) for a refined subtyping of patients with HF.

**Methods:**

We utilised linked EHR from primary and secondary care, sourced from the Clinical Practice Research Datalink (CPRD) Aurum, which encompassed health data of over 30 million individuals in the UK. Individuals aged 35 and above with incident reports of HF between January 1, 2005, and January 1, 2018, were included. We proposed a Transformer-based approach to cluster patients based on all clinical diagnoses, procedures, and medication records in EHR. Statistical machine learning (ML) methods were used for comparative benchmarking. The models were trained on a derivation cohort and assessed for their ability to delineate distinct clusters and prognostic value by comparing one-year all-cause mortality and HF hospitalisation rates among the identified subgroups in a separate validation cohort. Association analyses were conducted to elucidate the clinical characteristics of the derived clusters.

**Findings:**

A total of 379,108 patients were included in the HF subtyping analysis. The Transformer-based approach outperformed alternative methods, delineating more distinct and prognostically valuable clusters. This approach identified seven unique HF patient clusters characterised by differing patterns of mortality, hospitalisation, and comorbidities. These clusters were labelled based on the dominant clinical features present at the initial diagnosis of HF: early-onset, hypertension, ischaemic heart disease, metabolic problems, chronic obstructive pulmonary disease (COPD), thyroid dysfunction, and late-onset clusters. The Transformer-based subtyping approach successfully captured the multifaceted nature of HF.

**Interpretation:**

This study identified seven distinct subtypes, including COPD-related and thyroid dysfunction-related subgroups, which are two high-risk subgroups not recognised in previous subtyping analyses. These insights lay the groundwork for further investigations into tailored and effective management strategies for HF.

**Funding:**

10.13039/501100000274British Heart Foundation, European Union - Horizon Europe, and Novo Nordisk Research Centre Oxford.

**Research in context**

**Evidence before this study**

We searched PubMed, medRxiv, bioRxiv, and arXiv for English-language studies published from January 1, 2000, to December 31, 2024. Our keywords included “machine learning”, “deep learning”, “heart failure”, “subtype”, “subgroup”, and “cluster”. The majority of these studies on heart failure (HF) subtyping were conducted on small cohorts. A few utilised large, nationally representative cohorts, such as the Swedish Heart Failure Registry and the Health Improvement Network (THIN). Existing methods for clustering patients with HF based on electronic health records (EHR) data were predominantly limited to expert-guided methods utilising statistical machine learning (ML) techniques or the manual selection of risk factors. Most research identified several cardiometabolic-related HF subtypes.

**Added value of this study**

This study proposes a Transformer-based deep learning (DL) approach to subtype patients with HF using high-dimensional longitudinal EHR. We introduced a training paradigm leveraging the extensive sequence modelling capabilities of the Transformer model to identify patient clusters from EHR. Our results demonstrate that DL models have advantages over statistical ML models in terms of cluster distinctiveness and prognostic significance, highlighting the utility of DL for subtyping research. Furthermore, our method uncovered clusters associated with chronic obstructive pulmonary disease and thyroid dysfunction—two subgroups associated with higher mortality risk that had not been identified in previous HF subtyping analyses.

**Implications of all the available evidence**

Our analyses were based on large-scale, linked EHR encompassing over 30 million individuals in the UK to identify and validate these HF subtypes. These subtypes varied in terms of mortality, hospitalisation, disease associations, and post-HF risk profiles. Our proposed model was reliably validated across different cohorts, demonstrating its robustness.

## Introduction

Heart failure (HF) is a complex clinical syndrome with known heterogeneity in its presentation and progression. In clinical practice, identifying subtypes of HF primarily involves measuring left ventricular ejection fraction (LVEF) and categorising patients into those with reduced ejection fraction (HFrEF) and preserved ejection fraction (HFpEF).^1^ However, LVEF’s variability and limited reflection of HFpEF pathophysiology have raised concerns about its reliability and comprehensiveness for characterising HF.^2^^,^^3^ The availability of detailed medical histories in large-scale electronic health records (EHR) together with advanced modelling approaches have paved the way for a shift from unidimensional classifications of HF towards one that is more reflective of the underlying mechanisms of the disease and its progression.^4^

Recent machine learning (ML) research on large-scale EHR has revealed distinct HF subgroups, demonstrating varied mortality risks and suggesting potential differences in the underlying biological mechanisms of ML-identified subgroups.^5^^,^^6^ However, many of these studies focus on a small set of predefined clinical factors when conducting subtyping, usually those tied to known prognostic outcomes (particularly mortality). This approach limits the depth of exploration into the heterogeneity of HF and potentially overlooks important but less apparent pathological features. Considering the complexity of HF, it is crucial for subtyping models to transcend expert-driven modelling, offering a broader range of data-driven insights into HF classification. Besides feature selection, the prevalent use of statistical ML models for HF subtyping in prevailing studies has its own set of limitations. These models typically analyse HF-related factors in isolation and lack robust mechanisms to capture the relationships between different factors.^7^ Given that HF often develops alongside multiple existing comorbidities, modelling approaches that fail to account for this complexity are insufficient.

To address these limitations, deep learning (DL) data-driven modelling approaches have emerged as a promising alternative. Specifically, the Transformer^8^ model, initially designed for textual data analysis, has proven its adaptability on a variety of forms of sequential data, including longitudinal EHR.^9^^,^^10^ The model has shown advanced predictive capabilities across a range of clinical outcome prediction tasks by utilising EHR sequences, surpassing both expert-guided and traditional ML approaches.^9^^,^^11^ Leveraging the advanced EHR modelling capabilities of Transformer, we employed it to identify distinct HF subtypes using high-dimensional, longitudinal UK EHR data.

## Methods

### Data

We used Clinical Practice Research Datalink (CPRD) Aurum as the data source for this study.^12^ CPRD includes extensive patient EHR from UK general practices (GPs), accounting for about 20% of the UK population. This includes demographics, diagnoses, prescriptions, tests, and lifestyle data. The dataset is linked with Hospital Episode Statistics (HES) and Office for National Statistics (ONS) for secondary care and mortality data respectively. It has been extensively validated for epidemiological research, ensuring good completeness and representativeness.^13^

### Patient population

We divided the CPRD cohort into a derivation and validation cohort. Specifically, we selected patients from a random set of 80% of GPs for derivation, and the remaining 20% for validation. This ensures models are derived and validated across cohorts representing diverse geographic and socioeconomic characteristics (Supplementary Table S1), enhancing the robustness of our findings.^14^

We selected patients who were aged 35 years and older, with incident reports of HF between Jan 1, 2005, and Jan 1, 2018. Selection criteria included adherence to CPRD quality standards, eligibility for CPRD and HES linkage, and a minimum of 12 months of registration with their GPs. Incident HF was defined as the first recorded HF event in primary or secondary care. HF was identified using diagnostic codes based on previously validated code lists.^15^^,^^16^

### Ethics

The CPRD Group holds ethical approval from a National Research Ethics Service Committee for all purely observational studies conducted using CPRD. This study received scientific approval from the CPRD Independent Scientific Advisory Committee (protocol number: 20_095).

### Clustering algorithms

Clustering patients based on their medical records involves vectorisation, the process of transforming EHR data into compressed vectors that represent their medical histories. To train these models and derive meaningful clusters, we utilised patient EHR exclusively from the period prior to their HF diagnosis.

Our proposed DL approach employed a Transformer-based architecture, which incorporates specialised layers to encode clinical encounters along with temporal and visit-specific information (i.e., age, year, and visit number).^9^ Details of the input structure for the model can be found in Supplementary Text: Processing of Electronic Health Records. The model uses its “self-attention” mechanism to dynamically capture the importance of each medical encounter in the context of the patient’s entire medical history.^8^ This allows for more nuanced modelling of the evolving and dynamic nature of patient health. We trained the Transformer model using an unsupervised objective known as contrastive learning,^17^ an effective approach for refining sequence representations across various data types (e.g., text, medical images).^18^ Contrastive learning operates on the principle of drawing similar patients (in terms of medical history and risk profiles) closer and distancing dissimilar ones within the space of all patient vector representations. This optimisation approach produces patient embeddings that are both cohesive and discriminative, thereby enhancing our ability to capture subtle patterns in patient trajectories. For further information regarding the specifics of our training methodology and the implementation of contrastive learning, please refer to the Supplementary Text: Transformer model training details. To derive patient representation vectors for clustering, we averaged the first and final layers of model vector outputs for each patient, as this pooling method combines the generalised insights about a patient’s EHR.^19^ Following vectorisation, K-means was used for clustering.

To directly compare the findings from the proposed approach with more conventional ML approaches, we additionally employed the Term Frequency-Inverse Document Frequency (TF-IDF)^20^ method. TF-IDF quantifies the significance of medical encounters by their frequency within the entire patient cohort, thereby reducing the potential biases of manual feature selection. This method has been validated by prior HF subtyping studies for its effectiveness in processing EHR.^21^ Details of our implementation of TF-IDF on EHR data are provided in the Supplementary Text: TF-IDF. A potential limitation of TF-IDF is that it takes a cross-sectional snapshot of medical history at baseline, thereby overlooking the temporal interplay between various HF-related factors. Following vectorisation, the K-Means model was employed to group patients with similar vectors into clusters.

### Determination of the optimal number of clusters

We utilised the prediction strength method to determine the optimal number of clusters for each approach.^22^ Prediction strength measures how consistently a given pair of observations is classified into the same cluster across different data subsets (e.g., training and testing subsets). Subsets were defined by a five-fold cross-validation strategy solely on the derivation cohort. Utilising the prediction strength metric elucidates the conditions under which the approach operates most reliably. Additional information is provided in Supplementary Text: Prediction strength calculation.

### Cluster analysis

Upon training and deriving the optimal number of clusters within the derivation cohort, the models were applied to the validation cohort to establish and analyse patient clusters. We compared the clustering performance of the Transformer and TF-IDF methods based on the distinctiveness of clusters and their prognostic value.

To evaluate the distinctiveness of clusters, we examined how effectively the models separate patients with different characteristics within the vector space representing their medical histories. This was quantified using two metrics: the silhouette score,^23^ which measures the cohesiveness and separation of clusters, and the Calinski-Harabasz score,^24^ which evaluates the variance ratio between and within clusters. To evaluate the prognostic relevance of the clustering approaches, we conducted survival analyses. We utilised cumulative incidence plots with the Kaplan–Meier estimator to visualise one-year all-cause mortality and hospitalisation rates after HF onset across the identified clusters. Clusters exhibiting more distinct incidence patterns were considered to more meaningfully differentiate between prognostically diverse subgroups. Incidence rates were calculated with 95% confidence intervals using Greenwood’s exponential formula.^25^ The modelling approach with a higher cluster distinctiveness and greater prognostic diversity provided was considered better in delineating the heterogenous HF population.

To further understand the clinical implications of the clusters identified by the optimal approach, we performed three sets of analyses. First, we examined the variance in the prevalence of all recorded comorbidities, procedures, and medications prior to the first recorded diagnosis of HF among the clusters. We highlighted variables with at least a 30% variance between the most and least prevalent clusters and presented them in a covariance matrix. Second, we presented baseline demographic data for patients in each cluster, with missing measurements imputed through Multivariate Imputation by Chained Equations (Supplementary Text: Imputation). Additionally, we showed the distribution of HFrEF and HFpEF within each cluster. As LVEF measurements are not recorded in the CPRD, patients were classified as HFrEF or HFpEF based solely on diagnostic codes from primary and secondary care explicitly indicating these subtypes. Patients without a diagnostic code specifying the LVEF subtype were categorised as unspecified HF. The classification process was based on a previously validated code list (Supplementary Table S2).^26^ Third, we assessed the one-year incident rates of common HF comorbidities and sequelae following the diagnosis of HF to understand the disease progression trajectories in different patient clusters. As this analysis investigated incident disease associations following HF, we excluded patients who had developed these conditions before their HF diagnosis. Results were graphically presented as cumulative incidence plots.

### Role of funders

The funders had no impact on the study design, data collection, data analysis, interpretation of the results, and the writing of the manuscript.

## Results

Overall, 379,108 individuals with HF, with a mean age of 77 years (SD: 12.4) and a roughly equal sex distribution, were included in the study (310,723 in the derivation cohort and 68,385 in the validation cohort) (Supplementary Fig. S1 and Table S1).

### Cluster identification

Using the prediction strength metric, we identified seven and four as the optimal number of clusters for the Transformer and statistical ML approaches, respectively (Supplementary Fig. S2). The proportion of patients in each cluster for both approaches is shown in Supplementary Fig. S3.

### Cluster analysis

In terms of cluster distinctiveness, the Silhouette and the Calinski-Harabasz score analyses were consistent in showing that the clusters identified by the Transformer model exhibited a higher degree of separation than those identified by the TF-IDF model (Table 1).

**Table 1:** Comparative analysis of cluster distinctiveness using Silhouette and Calinski-Harabasz scores.

Model/Score	TF-IDF	Transformer
Silhouette	0.05	0.36
Calinski-Harabasz	2191	23,371

The Silhouette score, ranging from −1 (poor fit) to 1 (excellent fit), measures how closely a vector matches its own cluster compared to others. The Calinski-Harabasz Index, with values starting from zero upwards, evaluates cluster distinctiveness by comparing between-cluster separation to within-cluster closeness. Higher values for both metrics indicate better clustering. TF-IDF stands for term frequency-inverse document frequency.

**Fig. 1:**
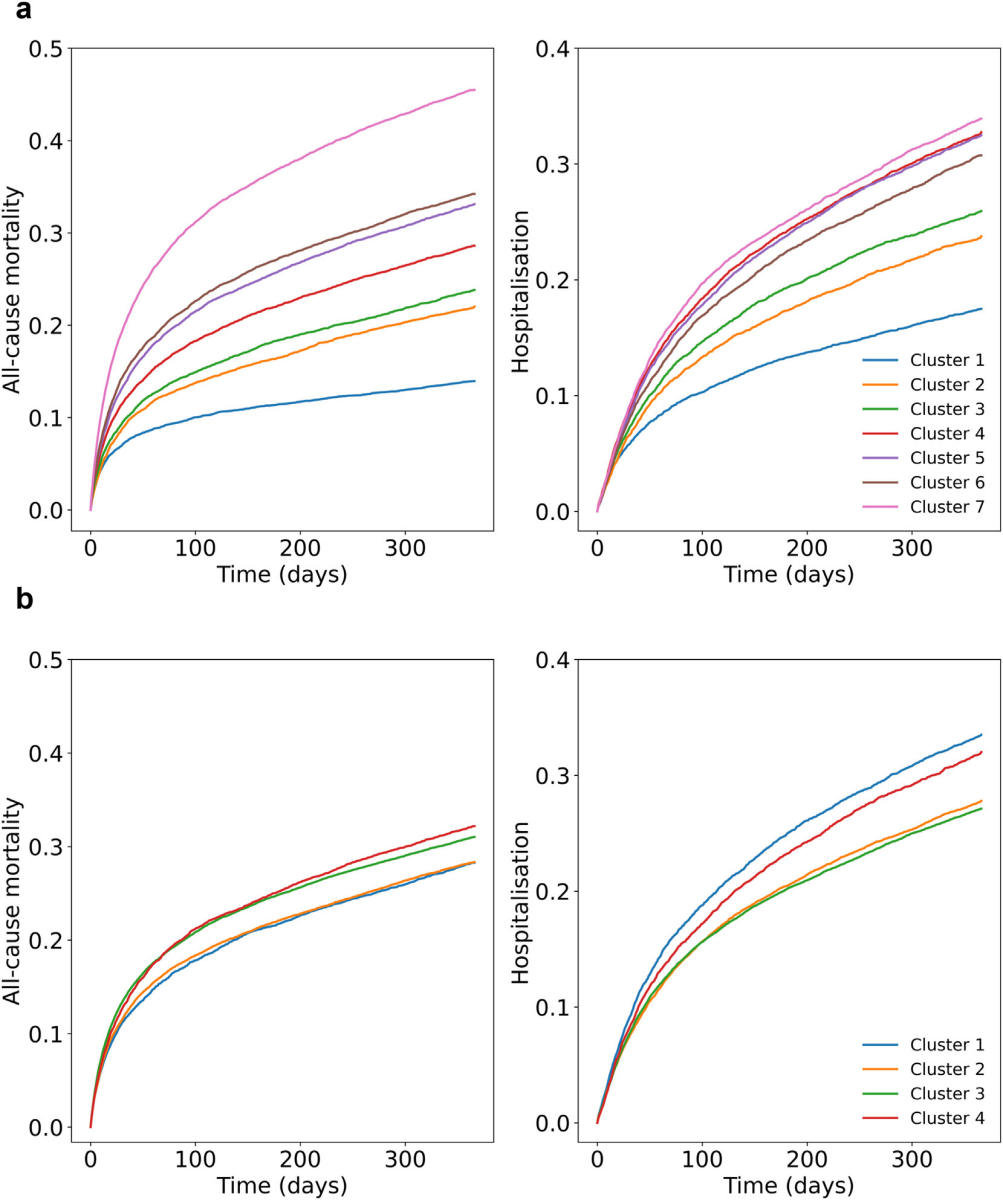
**Risk of all-cause mortality and heart failure hospitalisation by clusters identified.** (a) Transformer model. (b) Term frequency-inverse document frequency.

In terms of prognostic relevance, the clusters identified by the Transformer model displayed markedly distinct mortality patterns when compared to those identified through the TF-IDF method (Fig. 1). The one-year all-cause mortality ranged from 0.14 (95% CI 0.13–0.15) to 0.45 (95% CI 0.44–0.46) in clusters identified by the Transformer model, which was larger than the range observed with TF-IDF method, spanning from 0.28 (95% CI 0.27–0.29) to 0.32 (95% CI 0.31–0.33). Furthermore, HF hospitalisation curves from the Transformer model exhibited a greater separation between clusters, ranging from 0.17 (95% CI 0.17–0.18) to 0.34 (95% CI 0.33–0.35), compared to a range of 0.27 (95% CI 0.27–0.28) to 0.33 (95% CI 0.32–0.35) in the TF-IDF derived clusters. Additionally, previous studies have reported only modest differences in survival among patients with HFrEF and HFpEF,^27^ and we observed a similar pattern in the CPRD (Supplementary Fig. S4).

These findings suggest that the Transformer model more effectively captures complex interactions among HF risk factors, enabling the identification of patient subgroups with distinct risk profiles. Additionally, the Transformer-based approach demonstrated consistent prognostic separation across different cohorts, underscoring its robustness (Supplementary Fig. S5 and Table S3).

### Characterisation of transformer-identified clusters

We analysed the baseline demographic characteristics of HF clusters identified by the optimal approach, Transformer-based model, as depicted in Table 2. Clusters 1 and 7 exhibited the youngest and oldest mean ages, respectively. Sex distribution varied across clusters, with clusters 1 and 3 consisting mostly of men, and clusters 6 and 7 were predominantly composed of women. Sex-disaggregated demographic data can be found in the Supplementary Tables S4 and S5.

**Table 2:** Baseline characteristics of Transformer-identified clusters.

Cluster	1	2	3	4	5	6	7
**N**	8507	6454	8642	10,864	11,625	10,553	11,740
Mean age (in years) (SD)	61.8 (11.9)	78.9 (8.7)	78.4 (9.9)	75.3 (11.0)	77.0 (10.4)	82.0 (10.1)	87.8 (6.5)
Sex							
Male (%)	5689 (66.9)	3367 (52.2)	5798 (67.1)	6556 (60.3)	5896 (50.7)	3543 (33.6)	4114 (35.0)
Female (%)	2818 (33.1)	3087 (47.8)	2844 (32.9)	4308 (39.7)	5729 (49.3)	7010 (66.4)	7626 (65.0)
**Ethnicity**							
White (%)	7560 (88.9)	5959 (92.3)	8162 (94.4)	9169 (84.4)	10,959 (94.3)	9864 (93.5)	11,130 (94.8)
Black (%)	225 (2.6)	107 (1.7)	60 (0.7)	452 (4.2)	115 (1.0)	148 (1.4)	49 (0.4)
Asian (%)	216 (2.5)	103 (1.6)	218 (2.5)	888 (8.2)	272 (2.3)	255 (2.4)	118 (1.0)
Other or unknown (%)	506 (5.9)	285 (4.4)	202 (2.3)	355 (3.3)	279 (2.4)	286 (2.7)	443 (3.8)
**Socioeconomic status quintile**							
Quintile 1 (least deprived) (%)	2085 (24.5)	1796 (27.8)	2294 (26.5)	2135 (19.7)	2390 (20.6)	2615 (24.8)	3205 (27.3)
Quintile 2 (%)	1696 (19.9)	1354 (21.0)	1761 (20.4)	1961 (18.1)	2096 (18.0)	2129 (20.2)	2532 (21.6)
Quintile 3 (%)	1743 (20.5)	1385 (21.5)	1769 (20.5)	2186 (20.1)	2400 (20.6)	2274 (21.5)	2507 (21.4)
Quintile 4 (%)	1440 (16.9)	1043 (16.2)	1400 (16.2)	2090 (19.2)	2131 (18.3)	1773 (16.8)	1827 (15.6)
Quintile 5 (most deprived) (%)	1543 (18.1)	876 (13.6)	1418 (16.4)	2492 (22.9)	2608 (22.4)	1762 (16.7)	1669 (14.2)
**Smoking status∗**							
Smoker (%)	2474 (29.1)	746 (11.6)	1189 (13.8)	1577 (14.5)	2694 (23.2)	1242 (11.8)	1235 (10.5)
Ex-smoker (%)	3272 (38.5)	2802 (43.4)	4369 (50.6)	5004 (46.1)	6205 (53.4)	4747 (45.0)	4699 (40.0)
Non-smoker (%)	2761 (32.5)	2906 (45.0)	3084 (35.7)	4283 (39.4)	2726 (23.4)	4564 (43.2)	5806 (49.5)
**Measurements**							
Mean SBP (SD)	132.8 (18.5)	137.8 (18.9)	132.5 (18.3)	135.1 (18.5)	132.9 (18.4)	133.5 (18.7)	133.3 (19.0)
Mean DBP (SD)	79.5 (11.6)	76.3 (11.2)	73.8 (10.7)	73.4 (10.9)	74.8 (11.1)	74.1 (10.9)	74.2 (10.9)
Mean BMI (SD)	28.9 (6.2)	28.6 (5.9)	27.5 (5.0)	30.6 (6.4)	27.7 (6.4)	28.0 (6.4)	25.0 (5.0)

%: percent; SD: standard deviation; BMI: body mass index; SBP: systolic blood pressure; DBP: diastolic blood pressure; Values with ∗ have missing data and were imputed. Missingness: Systolic blood pressure (8.6%), Diastolic blood pressure (8.6%), BMI (41.1%), Smoking Status (22.6%).

**Fig. 2:**
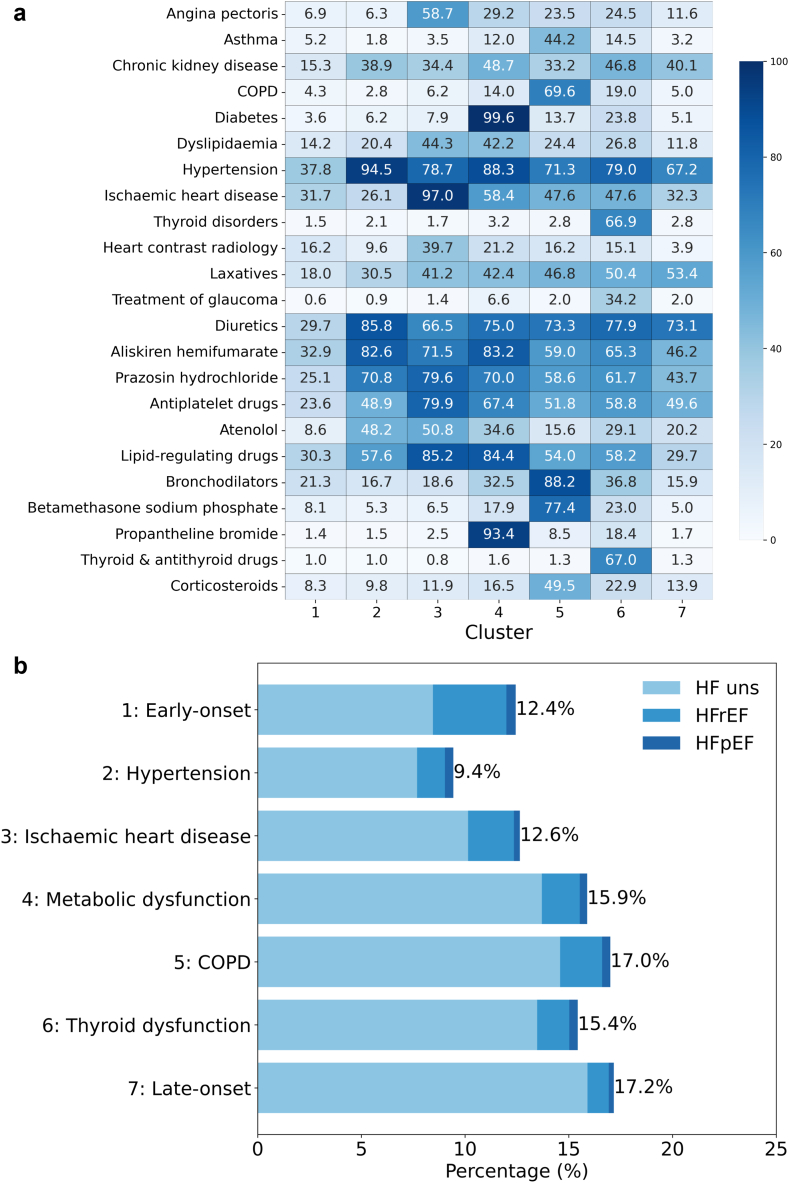
**(a) Associations between heart failure (HF) clusters and clinical characteristics.** Each cell represents the proportion of patients within a specific cluster who experienced certain conditions at the index time. (b) Patient distribution of HF clusters. This bar chart shows the percentage of patients in each cluster. Each colour represents the proportion of patients categorised into HF with reduced ejection fraction (HFrEF), HF with preserved ejection fraction (HFpEF), and unspecified HF (HF uns). COPD: Chronic obstructive pulmonary disease.

Fig. 2A presents the association heatmap between clusters and clinical characteristics that significantly varied among the clusters. It revealed that clusters 1 and 7 are characterised by fewer comorbidities. In contrast, clusters 2 through 6 exhibited distinct clinical profiles, marked by specific comorbid conditions. The covariance matrix for TF-IDF and LVEF-based subtypes is presented in the Supplementary Fig. S6.

Fig. 2B shows the proportion of patients for each cluster, as well as the proportions of LVEF-based HF subtypes within each cluster. Each cluster is named based on its prominent characteristics. The distribution of LVEF-based subgroups, encompassing both HFrEF and HFpEF, was similar across all clusters, suggesting that factors beyond LVEF played a pivotal role in the clustering process.

Supplementary Table S6 further elaborates on these findings, providing a concise summary of the principal clinical traits associated with each cluster.

### Incidence of post-HF comorbidities

**Fig. 3:**
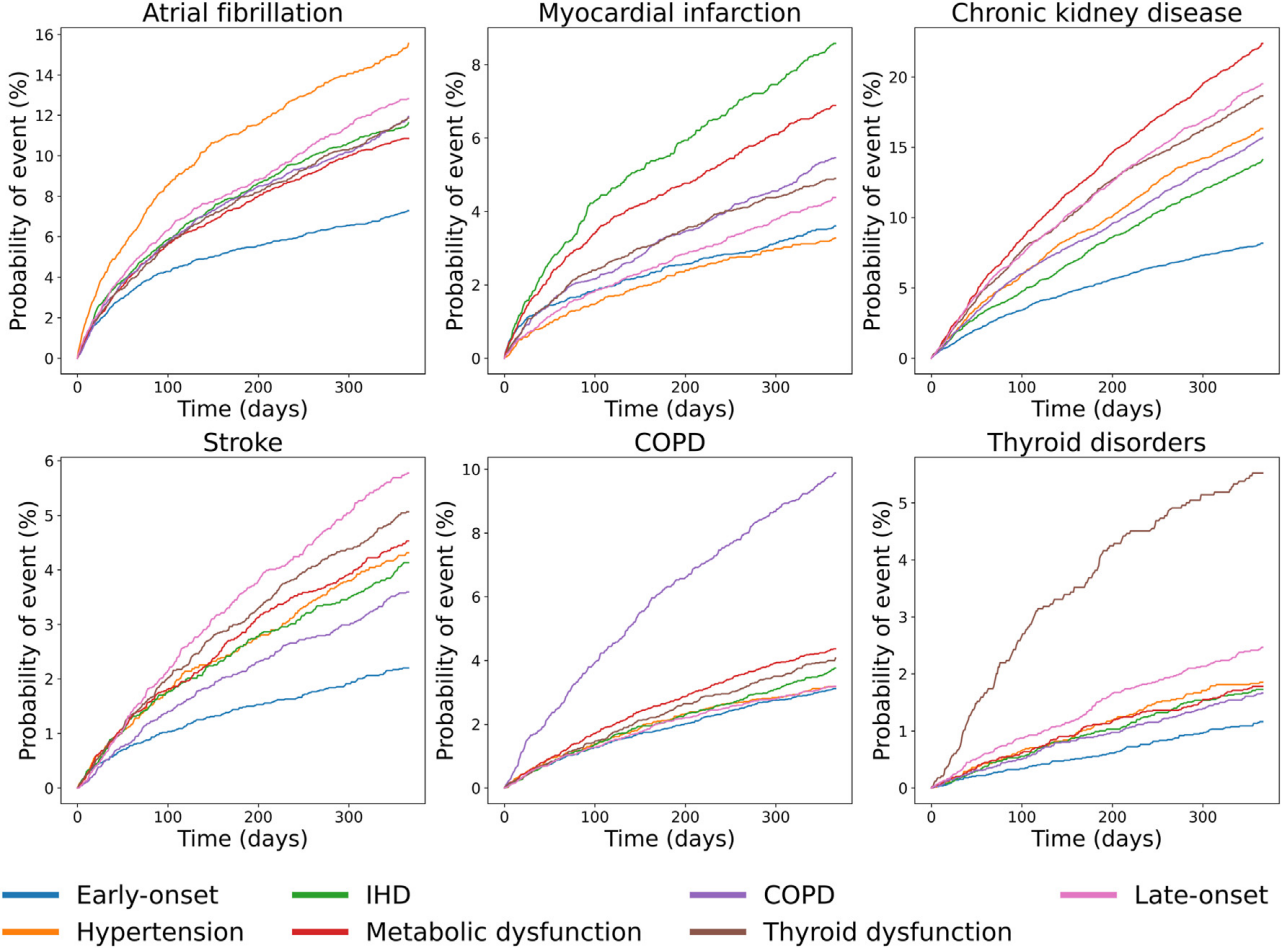
**Cumulative incidence of six comorbid conditions within a one-year follow-up period for seven heart failure subtypes.** Each line within the graphs corresponds to a different HF subtype, showing the risk of comorbid event occurrence over time. COPD: Chronic obstructive pulmonary disease.

Incidence analysis across various patient clusters revealed distinct patterns in the development of diseases after incident HF (Fig. 3). The early-onset cluster typically exhibited the lowest risk for most sequelae. The hypertension-related cluster demonstrated the highest risk for atrial fibrillation (AF), while the ischaemic heart disease-related cluster showed a particularly high risk for developing myocardial infarction. The metabolic dysfunction cluster was most prone to chronic kidney disease (CKD), and the late-onset cluster generally faced higher risks for multiple events, including AF and CKD, with stroke presenting the highest risk among all clusters. Notably, patients in the COPD and thyroid dysfunction-related clusters, who had not developed these conditions before HF diagnosis, are at the highest subsequent risk of developing them after HF.

## Discussion

Our study derived and validated a Transformer-based approach for data-driven clustering of patients with HF utilising routine EHR. We identified seven distinct HF clusters with significantly different mortality, hospitalisation rates, and comorbidity patterns. We labelled each cluster based on predominant characteristics at the index time as follows: (1) early onset, (2) hypertension, (3) ischaemic heart disease, (4) metabolic dysfunction, (5) COPD, (6) thyroid dysfunction, and (7) late-onset. Notably, each cluster exhibited unique trajectories in terms of the incidence of post-HF comorbidities and sequelae.

Consistent with previous HF subtyping studies, we identified clusters associated with metabolic dysfunction, age-related subtypes,^6^ and those primarily composed of patients with hypertension or IHD^5^^,^^28^—factors that are widely studied as major contributors to the development and progression of HF.^29^^,^^30^ Beyond these previously recognised subtypes, our study identified two additional subgroups characterised by COPD and thyroid dysfunction. While these conditions have long been recognised as secondary risk factors for HF,^31^^,^^32^ our DL-based approach identified them as distinct pathophysiological entities with unique clinical trajectories and prognoses compared to other subgroups. These findings suggest that COPD and thyroid dysfunction might not only exacerbate HF but also play critical roles in its development and progression, likely through distinct pathophysiological mechanisms.^33^

Our Transformer-based DL approach demonstrates a methodological refinement in the use of EHR data for HF subtyping. By leveraging contrastive learning, the model captures the complexity of multimodal EHR data, enabling the identification of subtypes based on all available clinical features. Unlike traditional disease clustering approaches, this method does not rely on predefined feature selection or expert-driven assumptions, making it more data-driven and reflective of patient heterogeneity. When compared with other approaches capable of encoding comprehensive EHR information, such as TF-IDF, our Transformer model identified subtypes with more distinct patterns in mortality and hospitalisation outcomes. TF-IDF delineated four clusters, but they lacked the clear prognostic separation seen in Transformer-derived subtypes. This suggests an enhanced ability of the Transformer model to capture nuanced clinical representations and prognostic differences.

Importantly, our data-driven clustering pipeline could offer new opportunities for tailored subgroup-specific treatment strategies. For instance, while beta-blockers are considered a first-line treatment for HF, it is well known that they must be carefully selected and dosed in patients with COPD to prevent exacerbating respiratory symptoms.^34^ Similarly, patients with HF and thyroid dysfunction may require different treatment approaches that specifically address thyroid hormone levels, as these can significantly impact cardiovascular health and overall HF management.^32^ In general, sensitive segmentation of these high-risk patient groups underscores the need for a more refined understanding of the unique characteristics of each subgroup. This leads to critical research questions: Are these subgroups fundamentally different from other patients with HF? Does the varied nature of these groups imply a need for specifically tailored management strategies? Are there more effective HF management strategies for these clusters compared to standard approaches? Such questions pave the way for future observational and randomised studies, inviting deeper exploration into how personalised treatment strategies could better address the specific needs of patients within these newly identified clusters, potentially leading to improved outcomes in heart failure management.

Lastly, we showed that the subgroup membership can be a proxy marker for downstream risk. For example, membership in the COPD subgroup can indirectly imply that the patient is at a higher risk of mortality than someone in the other subgroups. This indicates that the Transformer model transcends the mere reflection of historical medical data by providing prognostic insight into the future health trajectory of patients. It adeptly pinpoints those at a higher risk of developing certain comorbidities, even without using post-HF EHR data for training and clustering. This implies that patients clustered with these defining conditions may have pathophysiological pathways aligned with those who have already been diagnosed, highlighting the importance of building models that are able to capture these latent factors for the management and treatment of HF. By grouping patients based on shared phenotypic characteristics beyond single diagnoses, this approach captures broader biologically relevant traits, reduces reliance on potentially inaccurate diagnostic codes, improves statistical power for detecting genetic associations (particularly rare variants) through larger yet phenotypically meaningful cohorts.^35^ In this way, our study lays the groundwork for future discoveries, particularly in genetic research such as Genome-Wide Association Studies (GWAS), by identifying phenotypically meaningful subgroups that may aid in uncovering shared genetic or molecular pathways. These findings provide a foundation for advancing our understanding of HF and its subtypes, enabling further exploration of the biological mechanisms underlying disease progression and informing future precision medicine approaches.

### Strengths and limitations

We developed a Transformer-based tool for HF subtyping using routine multimodal EHR and conducted a comprehensive analysis comparing DL and statistical ML approaches on linked UK EHR. To ensure the generalisability of our findings, all analyses were conducted on a validation cohort from separate GPs, distinct from the derivation cohort. A key strength of our study is the use of the CPRD, a vast and representative EHR database, which ensures that our findings are reflective of real-world trends across primary and secondary care. Additionally, the scalability of the Transformer-based framework allows it to be adapted not only to other EHR datasets, such as the UK Biobank,^36^ but also to other subtyping research questions beyond HF, enabling its application across different diseases and conditions. Furthermore, the model can be easily extended to integrate additional data modalities,^37^ including genetic, proteomic, or imaging data, offering more comprehensive stratification.

Our study also has some limitations. First, although CPRD is a large and representative dataset, the clusters were derived and validated entirely within this single dataset. Expanding the application to datasets from other healthcare systems or populations would further enhance the robustness and generalisability of our findings. Second, our analysis relied solely on EHR data and did not include other data sources, such as cardiac imaging, genetic information. The absence of these data limits the inclusion of protein-based and imaging biomarkers in understanding the underlying mechanisms and progression of HF. Third, while our findings demonstrate distinct HF subgroups with varied clinical trajectories and prognoses, further work is required to translate these insights into clinical applications. This could include genetic analyses to identify subgroup-specific biomarkers for therapeutic discovery, and clinical trials to assess the effectiveness of tailored interventions within these clusters.

Our study advances HF subtyping by moving beyond traditional subtyping methods to a more nuanced, data-driven approach. By employing advanced DL techniques, we have delineated HF subtypes with distinct clinical profiles, advancing aetiological understanding of HF, paving the way for more personalised and effective HF management strategies. These findings underscore the potential of DL in transforming patient subgrouping, highlighting its role as an indispensable tool in the era of precision medicine.

## Contributors

ZF, MM, and KR conceived the research question. ZF, YL, and SR processed the data. ZF was responsible for deriving and implementing the models, analysing the results, and drafting the manuscript. SR supervised modelling, data analysis, and interpretation of results, and provided revisions of the manuscript. KR reviewed the manuscript and supervised the interpretation of the data. All authors reviewed, provided feedback on, and approved the final manuscript. ZF and SR verified the underlying data of the current study.

## Data sharing statement

Access to CPRD data, including UK Primary Care Data and linked datasets like Hospital Episode Statistics, requires protocol approval through CPRD’s Research Data Governance Process (https://www.cprd.com/research-applications). The code for model training is available on the GitHub (https://github.com/Zhengxian-Fan/HF-Subtyping).

## Declaration of interests

SR receives honoraria or consulting fees from BMJ Heart and Lucem Health and reports grants from Medical Research Council (MRC) and Oxford University Hospitals Trust. KR is currently supported by the UK Research and Innovation’s Global Challenge Research Fund (grant number: ES/P011055/1). Additionally, KR has previously received consulting fees from Medtronic CRDN and honoraria or fees from BMJ Heart, PLoS Medicine, AstraZeneca MEA Region, Medscape, and WebMD Medscape UK.

## References

[1] McDonagh T.A., Metra M., Adamo M. (2021). 2021 ESC guidelines for the diagnosis and treatment of acute and chronic heart failure. Eur Heart J.

[2] Lupón J., Gavidia-Bovadilla G., Ferrer E. (2018). Dynamic trajectories of left ventricular ejection fraction in heart failure. J Am Coll Cardiol.

[3] Triposkiadis F., Butler J., Abboud F.M. (2019). The continuous heart failure spectrum: moving beyond an ejection fraction classification. Eur Heart J.

[4] Banerjee A., Chen S., Fatemifar G. (2021). Machine learning for subtype definition and risk prediction in heart failure, acute coronary syndromes and atrial fibrillation: systematic review of validity and clinical utility. BMC Med.

[5] Ahmad T., Lund L.H., Rao P. (2018). Machine learning methods improve prognostication, identify clinically distinct phenotypes, and detect heterogeneity in response to therapy in a large cohort of heart failure patients. J Am Heart Assoc.

[6] Banerjee A., Dashtban A., Chen S. (2023). Identifying subtypes of heart failure from three electronic health record sources with machine learning: an external, prognostic, and genetic validation study. Lancet Digit Health.

[7] Mikolov T., Chen K., Corrado G. (2013). 1st international conference on learning representations, ICLR 2013 - workshop track proceedings.

[8] Vaswani A., Shazeer N., Parmar N. (2017). Advances in neural information processing systems.

[9] Li Y., Rao S., Solares J.R.A. (2020). BEHRT: transformer for electronic health records. Sci Rep.

[10] Devlin J., Chang M.-W., Lee K. (2019).

[11] Placido D., Yuan B., Hjaltelin J.X. (2023). A deep learning algorithm to predict risk of pancreatic cancer from disease trajectories. Nat Med.

[12] Wolf A., Dedman D., Campbell J. (2019). Data resource profile: clinical practice research Datalink (CPRD) Aurum. Int J Epidemiol.

[13] Jick S., Vasilakis-Scaramozza C., Persson R. (2023). Use of the CPRD Aurum database: insights gained from new data quality assessments. Clin Epidemiol.

[14] Hippisley-Cox J., Coupland C., Brindle P. (2017). Development and validation of QRISK3 risk prediction algorithms to estimate future risk of cardiovascular disease: prospective cohort study. BMJ.

[15] Conrad N., Verbeke G., Molenberghs G. (2022). Autoimmune diseases and cardiovascular risk: a population-based study on 19 autoimmune diseases and 12 cardiovascular diseases in 22 million individuals in the UK. Lancet.

[16] Denaxas S., Gonzalez-Izquierdo A., Direk K. (2019). UK phenomics platform for developing and validating electronic health record phenotypes: CALIBER. J Am Med Inform Assoc.

[17] Reimers N., Gurevych I. (2019). Proceedings of the 2019 conference on empirical methods in natural language processing and the 9th international joint conference on natural language processing (EMNLP-IJCNLP).

[18] Banville H., Chehab O., Hyvärinen A. (2021). Uncovering the structure of clinical EEG signals with self-supervised learning. J Neural Eng.

[19] Gao T., Yao X., Chen D. (2021). Proceedings of the 2021 conference on empirical methods in natural language processing.

[20] Sparck Jones Karen (1972). A statistical interpretation of term specificity and its application in retrieval. J Document.

[21] Nagamine T., Gillette B., Pakhomov A. (2020). Multiscale classification of heart failure phenotypes by unsupervised clustering of unstructured electronic medical record data. Sci Rep.

[22] Tibshirani R., Walther G. (2005). Cluster validation by prediction strength. J Comput Graphical Stat.

[23] Rousseeuw P.J. (1987). Silhouettes: a graphical aid to the interpretation and validation of cluster analysis. J Comput Appl Math.

[24] Calinski T., Harabasz J. (1974). A dendrite method for cluster analysis. Commun Stat Theory Methods.

[25] Bland J.M., Altman D.G. (1998). Statistics notes: survival probabilities (the Kaplan-Meier method). BMJ.

[26] Nakao Y.M., Nakao K., Nadarajah R. (2023). Prognosis, characteristics, and provision of care for patients with the unspecified heart failure electronic health record phenotype: a population-based linked cohort study of 95262 individuals. EClinicalMedicine.

[27] Owan T.E., Hodge D.O., Herges R.M. (2006). Trends in prevalence and outcome of heart failure with preserved ejection fraction. New Engl J Med.

[28] Nagamine T., Gillette B., Kahoun J. (2022). Data-driven identification of heart failure disease states and progression pathways using electronic health records. Sci Rep.

[29] Middlekauff H.R., Stevenson W.G., Stevenson L.W. (1991). Prognostic significance of atrial fibrillation in advanced heart failure. A study of 390 patients. Circulation.

[30] Dei Cas A., Fonarow G.C., Gheorghiade M. (2015). Concomitant diabetes mellitus and heart failure. Curr Probl Cardiol.

[31] Tavazzi L., Swedberg K., Komajda M. (2013). Clinical profiles and outcomes in patients with chronic heart failure and chronic obstructive pulmonary disease: an efficacy and safety analysis of SHIFT study. Int J Cardiol.

[32] Kannan L., Shaw P.A., Morley M.P. (2018). Thyroid dysfunction in heart failure and cardiovascular outcomes. Circ Heart Fail.

[33] Vargas-Uricoechea H., Bonelo-Perdomo A. (2017). Thyroid dysfunction and heart failure: mechanisms and associations. Curr Heart Fail Rep.

[34] Jabbour A., Macdonald P.S., Keogh A.M. (2010). Differences between beta-blockers in patients with chronic heart failure and chronic obstructive pulmonary disease. J Am Coll Cardiol.

[35] Garg M., Karpinski M., Matelska D. (2024). Disease prediction with multi-omics and biomarkers empowers case–control genetic discoveries in the UK Biobank. Nat Genet.

[36] Bycroft C., Freeman C., Petkova D. (2018). The UK Biobank resource with deep phenotyping and genomic data. Nature.

[37] Radford A., Kim J.W., Hallacy C. (2021). Proceedings of machine learning research.

