# Supplementary material for: Identification of heart failure subtypes using transformer-based deep learning modelling: a population-based study of 379,108 individuals
Source: eBioMedicine. 2025 Mar 19;114:105657. doi: 10.1016/j.ebiom.2025.105657 (PMC11979478; doi:10.1016/j.ebiom.2025.105657)
Supplement: Supplementary Text, Figs. S1–S6, and Tables S1–S6 [file mmc1.docx]

Supplementary Material

Table of Contents

[Supplementary Text 2](#_Toc170483724)

[Supplementary Tables 4](#_Toc170483725)

[Supplementary Figures 13](#_Toc170483726)

## Supplementary Text

**Processing of Electronic Health Records**

The Clinical Practice Research Datalink (CPRD) encompasses a range of coding systems for documenting medical information. Diagnoses in primary care and hospitals are recorded using both Med codes and ICD-10 codes. Procedures are categorised under OPCS-4 codes, and medication prescriptions are encoded using the Dictionary of Medicines and Devices (dm+d) and British National Formulary (BNF) codes. To ensure consistency across these diverse coding systems, we conducted a systematic mapping process. All diagnostic codes were aligned with ICD-10 codes at the fourth level of specificity. Similarly, for medications, dm+d codes were mapped to BNF codes at the section level and Virtual Therapeutic Moiety (VTM) using the NHS digital SNOMED CT dictionary. To create a comprehensive patient record, we concatenated all the mapped codes for diagnostic information, medication prescriptions, and medical procedures recorded up to the incident date of heart failure (HF). These sequences were enriched with additional context by including the patient's age and the year of each encounter.

**Transformer model training details**

An initial pre-training phase on the CPRD Electronic Health Record (EHR) dataset was undertaken, using a masked encounters modelling (MEM) objective. This involved randomly obscuring specific encounters and tasking the model to predict these hidden events, which aided the model in understanding the intricate interactions between different diseases and grasping the underlying distribution of the data. Following the pre-training phase, we represented patients by averaging the initial and final outputs of the model, blending broad insights from the patient’s EHR. We employed a contrastive learning objective, a proven method prominently utilised in the sequence modelling for refining representations,^18^ to tailor the model for patients with HF. Contrastive learning operates on the principle of drawing similar data points closer and distancing dissimilar ones within the model's embedding space. This optimisation approach makes clusters more cohesive internally and distinct from each other, enhancing the distinctiveness of clusters. Utilising EHR data of patients with HF, we generated positive pairs by bifurcating each patient’s EHR at a randomly chosen date between their GP registration and HF diagnosis. Encounters preceding the random date were paired with encounters post this date to form a positive sample, and future events from different patients were treated as negatives. This approach aids the model in discerning the unique trajectory of each patient's medical history, further distinguishing it from others. We also hid the age and year information of each patient at each visit during the model training phase, to prevent the model from identifying the differences by simply looking into ages.

Hyperparameters: Training parameters included a batch size of 64, a maximum sequence length of 250, and a learning rate set to 3e-5. The model was trained for ten epochs, with evaluations carried out every 500 steps. An early stopping criterion was set if the loss plateaued for five consecutive evaluations. We employed the multiple negative ranking loss as our chosen loss function.^35^

**Term Frequency-Inverse Document Frequency (TF-IDF)**

TF-IDF is a statistical method commonly used in natural language processing to evaluate the importance of terms within a document relative to a corpus. In this study, we adapted TF-IDF to process EHR data, treating EHR sequences as analogous to language model sequences. Each patient’s medical history was represented as a "document," where diagnostic codes served as "terms." This approach facilitated the transformation of EHR data into feature-rich vectors for clustering.

The TF-IDF vectorisation process consisted of the following steps:

1. The term frequency for a diagnostic code $t$ in a patient EHR $d$ was calculated as:

$$TF(t,d)=\frac{f_{t,d}}{\sum_{t^{'}\in d} f_{t^{'},d}}$$

where $f_{t,d}$is the count of $t$ in $d$, and the denominator is the total number of diagnostic codes in $d$.

1. The inverse document frequency of a diagnostic code $t$ was calculated as:

$$IDF(t)=\log\left( \frac{N}{1+n_{t}} \right)$$

where $N$ is the total number of patients in the cohort, and $n_{t}$is the number of EHR sequences containing $n_{t}$. Adding 1 to $n_{t}$​ avoids division by zero.

1. The TF-IDF score for each diagnostic code $t$ in a patient document $d$ was computed as:

$TF-IDF(t,d)=TF(t,d)\cdot IDF(t)$

This TF-IDF score quantified the relative importance of the diagnostic code for the patient within the context of the cohort. Each patient document was then converted into a TF-IDF vector, where each entry corresponded to the TF-IDF score of a diagnostic code.

**Prediction strength calculation**

The prediction strength is a measure used to determine the optimal number of clusters (k) by assessing the consistency with which pairs of data points are grouped together across training and validation sets, usually used in conjunction with five-fold cross-validation. In each iteration, 80% of the internal data was used as the training set $X_{tr}$, with the remaining 20% as the validation set $X_{te}$. The training set is used to train the representation model. After training, patients' EHRs were transformed into vector encodings and underwent a clustering algorithm to bothand $X_{te}$for k ranging from 2 to 10.

Given the clustering model $C\left( X_{tr},k \right)$ of the training set, we can generate a co-membership matrix $D\left[ C\left( X_{tr},k \right),X_{tr} \right]$ of [$n_{train}\times n_{train}]$ An element in the $ii^{'}$th position of this matrix is set to 1 if elements $i$ and $i'$of​ are $X_{tr}$ clustered together and 0 otherwise. Similarly, we can create a co-membership matrix for the test set. The prediction strength is the proportion of observation pairs that are assigned to the same clusters using the cluster centres from training and test set, and it selects the smallest of these proportions across the test clusters, thus providing a conservative measure of how well the training clusters predict the test clusters.

$$\mathrm{ps}(k)=\min_{1\leq j\leq k} \frac{1}{n_{kj}\left( n_{kj}-1 \right)}\sum_{i\neq i^{'}\in A_{kj}} D\left[ C\left( X_{\mathrm{tr}},k \right),X_{\mathrm{te}} \right]_{ii^{'}}$$

After completing this process for five iterations (5-fold cross-validation), the average prediction strength scores for cluster counts from 2 to 10 are calculated. The optimal number of clusters k is then chosen as the largest number for which the average prediction strength score exceeds a threshold, typically 0.90, indicating robust and stable clustering. This process ensures that the chosen number of clusters provides a reliable and consistent grouping across different subsets of the data, which is critical for the model's predictive power and generalisability.

**Imputation**

We extracted the most recent data recorded within 24 months prior to the initial diagnosis of HF. This included the latest measurements of blood pressure and body mass index (BMI), along with smoking status—categorised as current smoker, non-smoker, or ex-smoker—based on the most up-to-date records. Missing values for blood pressure, body mass index (BMI), and smoking status were imputed using the multiple imputation by chained equations (MICE) method over 10 iterations. The imputation models incorporated predictors including age, sex, ethnicity, index of multiple deprivation, atrial fibrillation, chronic kidney disease, chronic obstructive pulmonary disease, diabetes, dyslipidaemia, hypertension, ischemic heart disease, myocardial infarction, obesity, stroke, and thyroid disorders before incident HF. The imputed data were not utilised in statistical machine learning, deep learning modelling, or clustering; rather, the imputation was performed to present the baseline characteristics of the validation cohort.

## Supplementary Tables

**Supplementary Table 1. Baseline characteristics of derivation and validation cohorts.**

|  | **Derivation (N=310,723)** | **Validation (N=68,385)** |
| --- | --- | --- |
| Mean age (years) (SD) | 77.8 (12.3) | 77.3 (12.5) |
| Male (%) | 51.13% | 51.44% |
| **Ethnicity** | | |
| White (%) | 91.84% | 91.07% |
| Black (%) | 1.69% | 2.00% |
| Asian (%) | 3.03% | 3.33% |
| Other or unknown (%) | 3.45% | 3.59% |
| **Region** | |  |
| Northeast (%) | 4.49% | 3.82% |
| Northwest (%) | 19.33% | 22.08% |
| Yorkshire and the Humber (%) | 3.33% | 3.73% |
| East Midlands (%) | 3.53% | 2.06% |
| West Midlands (%) | 18.63% | 17.69% |
| East of England (%) | 3.71% | 4.39% |
| Southwest (%) | 15.17% | 13.45% |
| South Central (%) | 12.75% | 10.55% |
| London (%) | 11.43% | 13.57% |
| Southeast Coast (%) | 7.65% | 8.65% |
| **Socioeconomic Status Quintile** |  |  |
| Quintile 1 (%) | 24.16% | 18.88% |
| Quintile 2 (%) | 19.78% | 20.14% |
| Quintile 3 (%) | 20.86% | 19.50% |
| Quintile 4 (%) | 17.11% | 20.35% |
| Quintile 5 (%) | 18.09% | 21.13% |

**Supplementary Table 2. Clinical codes to identify HFrEF and HFpEF**

| **Code** | **Description** | **Category** | **Type** |
| --- | --- | --- | --- |
| 1661371000000112 | HFNEF - heart failure with normal ejection fraction | HFpEF | Med-code |
| 1647701000000118 | Heart failure with normal ejection fraction | HFpEF | Med-code |
| 2159198010 | Echocardiogram shows left ventricular diastolic dysfunction | HFpEF | Med-code |
| 1489358014 | Left ventricular diastolic dysfunction | HFpEF | Med-code |
| 2227501000000110 | Heart failure with preserved ejection fraction | HFpEF | Med-code |
| 7321121000006119 | Heart failure with preserved ejection fraction | HFpEF | Med-code |
| 970851000006119 | Echocardiogram shows normal left ventricular function | HFpEF | Med-code |
| 2534182016 | Echocardiogram shows normal left ventricular function | HFpEF | Med-code |
| 2159198010 | Echocardiogram shows left ventricular diastolic dysfunction | HFpEF | Med-code |
| 635781000006111 | Echocardiogram normal | HFpEF | Med-code |
| 262687015 | Echocardiogram normal | HFpEF | Med-code |
| 2159197017 | Echocardiogram shows left ventricular systolic dysfunction | HFrEF | Med-code |
| 784191000006110 | Impaired left ventricular function | HFrEF | Med-code |
| 411506018 | Impaired left ventricular function | HFrEF | Med-code |
| 299796018 | Ischaemic cardiomyopathy | HFrEF | Med-code |
| 9833971000006112 | Congestive heart failure stage C due to ischaemic cardiomyopathy | HFrEF | Med-code |
| 9834021000006114 | Systolic heart failure stage B due to ischaemic cardiomyopathy | HFrEF | Med-code |
| 9833991000006113 | Congestive heart failure stage B due to ischaemic cardiomyopathy | HFrEF | Med-code |
| 9834051000006117 | Systolic heart failure stage C due to ischaemic cardiomyopathy | HFrEF | Med-code |
| 2694523019 | Left ventricular cardiac dysfunction | HFrEF | Med-code |
| 216207010 | Left ventricular systolic dysfunction | HFrEF | Med-code |
| 2159197017 | Echocardiogram shows left ventricular systolic dysfunction | HFrEF | Med-code |
| 7510321000006115 | Asymptomatic left ventricular systolic dysfunction | HFrEF | Med-code |
| 7119761000006112 | Chronic left ventricular systolic dysfunction | HFrEF | Med-code |
| 7056281000006118 | Congestive heart failure due to left ventricular systolic dysfunction | HFrEF | Med-code |
| 1991651000006115 | Severe left ventricular systolic dysfunction | HFrEF | Med-code |
| 34556015 | Secondary dilated cardiomyopathy | HFrEF | Med-code |
| 6615901000006114 | Congestive dilated cardiomyopathy | HFrEF | Med-code |
| 7560351000006111 | X-linked dilated cardiomyopathy | HFrEF | Med-code |
| 7560361000006113 | Duchenne muscular dystrophy-associated dilated cardiomyopathy | HFrEF | Med-code |
| 3254291000006112 | Dilated cardiomyopathy secondary to dermatomyositis | HFrEF | Med-code |
| 6850191000006114 | Dilated cardiomyopathy associated with connective tissue disorder | HFrEF | Med-code |
| 7560331000006116 | Dilated cardiomyopathy 3B | HFrEF | Med-code |
| 2840701000006117 | Dilated cardiomyopathy secondary to rheumatoid arthritis | HFrEF | Med-code |
| 3706941000006119 | Dilated cardiomyopathy secondary to malignancy | HFrEF | Med-code |
| 3901271000006117 | Dilated cardiomyopathy secondary to infection | HFrEF | Med-code |
| 3219591000006115 | Dilated cardiomyopathy secondary to fungal myocarditis | HFrEF | Med-code |
| 2543761000006114 | Dilated cardiomyopathy secondary to myotonic dystrophy | HFrEF | Med-code |
| 2676691000006113 | Dilated cardiomyopathy secondary to phytanic acid storage disease | HFrEF | Med-code |
| 7475751000006117 | Ischaemic dilated cardiomyopathy due to coronary artery disease | HFrEF | Med-code |
| 2757771000006111 | Dilated cardiomyopathy secondary to peripartum heart disease | HFrEF | Med-code |
| 2530931000006112 | Dilated cardiomyopathy secondary to metazoal myocarditis | HFrEF | Med-code |
| 3687171000006119 | Dilated cardiomyopathy caused by drug | HFrEF | Med-code |
| 3075181000006114 | Dilated cardiomyopathy due to systemic sclerosis | HFrEF | Med-code |
| 3314361000006111 | Dilated cardiomyopathy secondary to Friedreich's ataxia | HFrEF | Med-code |
| 3452461000006110 | Dilated cardiomyopathy secondary to amyloidosis | HFrEF | Med-code |
| 7475781000006113 | Ischemic dilated cardiomyopathy due to coronary artery disease | HFrEF | Med-code |
| 3075191000006112 | Dilated cardiomyopathy secondary to scleroderma | HFrEF | Med-code |
| 3087841000006114 | Dilated cardiomyopathy secondary to systemic lupus erythematosus | HFrEF | Med-code |
| 3360091000006112 | Primary idiopathic dilated cardiomyopathy | HFrEF | Med-code |
| 3389851000006118 | Dilated cardiomyopathy secondary to bacterial myocarditis | HFrEF | Med-code |
| 3988271000006114 | Dilated cardiomyopathy secondary to polyarteritis nodosa | HFrEF | Med-code |
| 6850201000006112 | Dilated cardiomyopathy due to taurine deficiency | HFrEF | Med-code |
| 3138401000006112 | Dilated cardiomyopathy secondary to granuloma | HFrEF | Med-code |
| 3787161000006116 | Dilated cardiomyopathy secondary to neuromuscular disorder | HFrEF | Med-code |
| 7810851000006118 | Cardiac conduction disease with dilated cardiomyopathy and brachydactyly syndrome | HFrEF | Med-code |
| 7786921000006119 | Dilated cardiomyopathy with hypergonadotropic hypogonadism syndrome | HFrEF | Med-code |
| 3878171000006116 | Dilated cardiomyopathy secondary to nutritive deficiency | HFrEF | Med-code |
| 7476051000006113 | Foetal dilated cardiomyopathy | HFrEF | Med-code |
| 2991241000006116 | Dilated cardiomyopathy secondary to viral myocarditis | HFrEF | Med-code |
| 2631011000006118 | Dilated cardiomyopathy secondary to haemochromatosis | HFrEF | Med-code |
| 4193381000006112 | Dilated cardiomyopathy secondary to metabolic disorder | HFrEF | Med-code |
| 7475741000006119 | Dilated cardiomyopathy with genetic marker | HFrEF | Med-code |
| 3457111000006119 | Dilated cardiomyopathy secondary to glycogen storage disease | HFrEF | Med-code |
| 2631021000006114 | Dilated cardiomyopathy secondary to hemochromatosis | HFrEF | Med-code |
| 3053021000006110 | Dilated cardiomyopathy secondary to deficiency | HFrEF | Med-code |
| 2595681000006117 | Dilated cardiomyopathy secondary to sarcoidosis | HFrEF | Med-code |
| 3152591000006116 | Dilated cardiomyopathy secondary to protozoal myocarditis | HFrEF | Med-code |
| 3687131000006117 | Dilated cardiomyopathy secondary to drug | HFrEF | Med-code |
| 3859111000006117 | Dilated cardiomyopathy caused by alcohol | HFrEF | Med-code |
| 300040013 | Primary dilated cardiomyopathy | HFrEF | Med-code |
| 7791661000006111 | Wooly hair and palmoplantar keratoderma with dilated cardiomyopathy syndrome | HFrEF | Med-code |
| 3314371000006116 | Dilated cardiomyopathy secondary to Friedreich ataxia | HFrEF | Med-code |
| 2699901000006110 | Dilated cardiomyopathy secondary to radiation | HFrEF | Med-code |
| 3914551000006112 | Dilated cardiomyopathy secondary to mucopolysaccharidosis | HFrEF | Med-code |
| 7560341000006114 | DMD-associated dilated cardiomyopathy | HFrEF | Med-code |
| 2964231000006113 | Dilated cardiomyopathy secondary to muscular dystrophy | HFrEF | Med-code |
| 2699911000006113 | Dilated cardiomyopathy caused by radiation | HFrEF | Med-code |
| 6615851000006116 | Dilated cardiomyopathy | HFrEF | Med-code |
| 6615891000006110 | DCM - Dilated cardiomyopathy | HFrEF | Med-code |
| 2969451000006116 | Dilated cardiomyopathy secondary to sensitivity | HFrEF | Med-code |
| 7791641000006112 | Woolly hair and palmoplantar keratoderma with dilated cardiomyopathy syndrome | HFrEF | Med-code |
| 3553111000006118 | Dilated cardiomyopathy secondary to infiltration | HFrEF | Med-code |
| 2676711000006111 | Dilated cardiomyopathy secondary to Refsum disease | HFrEF | Med-code |
| 7476081000006117 | Fetal dilated cardiomyopathy | HFrEF | Med-code |
| 7850181000006112 | Sensorineural deafness with dilated cardiomyopathy syndrome | HFrEF | Med-code |
| 3966281000006113 | Dilated cardiomyopathy secondary to electrolyte deficiency | HFrEF | Med-code |
| 2529901000006111 | Dilated cardiomyopathy secondary to toxic reaction | HFrEF | Med-code |
| 2676701000006113 | Dilated cardiomyopathy secondary to Refsum's disease | HFrEF | Med-code |
| 3859091000006114 | Dilated cardiomyopathy secondary to alcohol | HFrEF | Med-code |
| 7688021000006114 | Dilated cardiomyopathy with ataxia syndrome | HFrEF | Med-code |
| I255 | Ischaemic cardiomyopathy | HFrEF | ICD-10 |
| I420 | Dilated cardiomyopathy | HFrEF | ICD-10 |

HFrEF: Heart failure with reduced ejection fraction; HFpEF: Heart failure with preserved ejection fraction

**Supplementary Table 3. Log-rank test results for differences in all-cause mortality and heart failure hospitalisation between the derivation and validation cohorts by cluster.**

| **Cluster** | **Mortality p-value** | **Hospitalisation p-value** |
| --- | --- | --- |
| 1 | 0.85 | 0.05 |
| 2 | 0.11 | 0.06 |
| 3 | 0.18 | 0.09 |
| 4 | 0.72 | 0.88 |
| 5 | 0.83 | 0.19 |
| 6 | 0.64 | 0.36 |
| 7 | 0.13 | 0.4 |

Clusters were identified by the Transformer model.

**Supplementary Table 4. Baseline characteristics of Transformer-identified clusters (Male patients only)**

| **Cluster** | **1** | **2** | **3** | **4** | **5** | **6** | **7** |
| --- | --- | --- | --- | --- | --- | --- | --- |
| N | 5,689 | 3,367 | 5,798 | 6,556 | 5,896 | 3,543 | 4,114 |
| Mean age (in years) (SD) | 61.6 (11.8) | 77.5 (8.9) | 76.8 (10.0) | 74.0 (10.9) | 76.0 (10.2) | 80.9 (10.2) | 86.8 (6.5) |
| **Ethnicity** | | | | | | | |
| White (%) | 5,078 (89.3) | 3,109 (92.3) | 5,482 (94.5) | 5,610 (85.6) | 5,557 (94.3) | 3,298 (93.1) | 3,906 (94.9) |
| Black (%) | 120  (2.1) | 61  (1.8) | 39  (0.7) | 238  (3.6) | 55  (0.9) | 66  (1.9) | 17  (0.4) |
| Asian (%) | 150  (2.6) | 53  (1.6) | 156 (2.7) | 503  (7.7) | 145 (2.5) | 103  (2.9) | 38  (0.9) |
| Other or unknown (%) | 341  (6.0) | 144  (4.3) | 121 (2.1) | 205  (3.1) | 139 (2.4) | 76  (2.1) | 153 (3.7) |
| **Socioeconomic status quintile** | | | | | | | |
| Quintile 1 (%) | 1,412 (24.8) | 938 (27.9) | 1,571 (27.1) | 1,364 (20.8) | 1,254 (21.3) | 934 (26.4) | 1,132 (27.5) |
| Quintile 2 (%) | 1,124 (19.8) | 711 (21.1) | 1,194 (20.6) | 1,224 (18.7) | 1,064 (18.0) | 771 (21.8) | 899 (21.9) |
| Quintile 3 (%) | 1,167 (20.5) | 727 (21.6) | 1,191 (20.5) | 1,335 (20.4) | 1,236 (21.0) | 713 (20.1) | 873 (21.2) |
| Quintile 4 (%) | 956 (16.8) | 546 (16.2) | 921 (15.9) | 1,222 (18.6) | 1,058 (17.9) | 566 (16.0) | 640 (15.6) |
| Quintile 5 (%) | 1,030 (18.1) | 445 (13.2) | 921 (15.9) | 1,411 (21.5) | 1,284 (21.8) | 559 (15.8) | 570 (13.9) |
| **Smoking Status^*^** | | | | | | | |
| Smoker (%) | 1,797 (31.6) | 418 (12.4) | 828 (14.3) | 968 (14.8) | 1,366 (23.2) | 403 (11.4) | 517 (12.6) |
| Ex-smoker (%) | 2,296 (40.4) | 1,824 (54.2) | 3,320 (57.3) | 3,575 (54.5) | 3,591 (60.9) | 2,072 (58.5) | 2,194 (53.3) |
| Non-smoker (%) | 1,596 (28.1) | 1,125 (33.4) | 1,650 (28.5) | 2,013 (30.7) | 939 (15.9) | 1,068 (30.1) | 1,403 (34.1) |
| **Measurements** | | | | | | | |
| Mean SBP (SD) | 133.0 (18.4) | 136.8 (18.2) | 131.5 (17.7) | 134.1 (18.1) | 131.6 (18.1) | 131.5 (17.6) | 131.6 (18.0) |
| Mean DBP (SD) | 79.8 (11.7) | 76.3 (11.3) | 73.8 (10.7) | 73.5 (10.8) | 74.5 (11.1) | 73.6 (11.1) | 73.1 (10.9) |
| Mean HR (SD) | 85.1 (24.1) | 78.2 (20.9) | 70.9 (18.2) | 78.2 (18.2) | 82.4 (18.1) | 75.8 (13.5) | 77.8 (16.6) |
| Mean BMI (SD) | 29.0 (5.8) | 28.5 (5.3) | 27.7 (4.7) | 30.4 (6.0) | 27.8 (5.7) | 27.7 (5.5) | 25.3 (4.4) |
| **Risk Factors** | | | | | | | |
| Atrial fibrillation (%) | 1,993 (35.0) | 1,806 (53.6) | 2,192 (37.8) | 2,363 (36.0) | 2,443 (41.4) | 1,743 (49.2) | 2,253 (54.8) |
| Chronic kidney disease (%) | 843 (14.8) | 1,274 (37.8) | 1,877 (32.4) | 3,072 (46.9) | 1,885 (32.0) | 1,598 (45.1) | 1,657 (40.3) |
| COPD (%) | 220  (3.9) | 113  (3.4) | 382 (6.6) | 976 (14.9) | 4,414 (74.9) | 706 (19.9) | 258 (6.3) |
| Diabetes (%) | 214  (3.8) | 219  (6.5) | 452 (7.8) | 6,529 (99.6) | 861 (14.6) | 906 (25.6) | 210 (5.1) |
| Dyslipidaemia (%) | 912 (16.0) | 687 (20.4) | 2,628 (45.3) | 2,816 (43.0) | 1,525 (25.9) | 1,034 (29.2) | 473 (11.5) |
| Hypertension (%) | 2,192 (38.5) | 3,141 (93.3) | 4,417 (76.2) | 5,699 (86.9) | 4,090 (69.4) | 2,733 (77.1) | 2,508 (61.0) |
| Ischaemic heart disease (%) | 2,057 (36.2) | 977 (29.0) | 5,649 (97.4) | 4,120 (62.8) | 3,086 (52.3) | 1,956 (55.2) | 1,448 (35.2) |
| Myocardial infarction (%) | 1,377 (24.2) | 405 (12.0) | 3,523 (60.8) | 2,368 (36.1) | 1,661 (28.2) | 1,073 (30.3) | 749 (18.2) |
| Obesity (%) | 591 (10.4) | 295  (8.8) | 462 (8.0) | 1,442 (22.0) | 691 (11.7) | 357 (10.1) | 143 (3.5) |
| Stroke (%) | 432  (7.6) | 672 (20.0) | 1,147 (19.8) | 1,380 (21.0) | 1,055 (17.9) | 795 (22.4) | 1,013 (24.6) |
| Thyroid disorders (%) | 46  (0.8) | 51  (1.5) | 61 (1.1) | 132  (2.0) | 97  (1.6) | 1,822 (51.4) | 70  (1.7) |

%: percent; SD: standard deviation; BMI: body mass index; SBP: systolic blood pressure; DBP: diastolic blood pressure; HR: Heart rate; COPD: Chronic obstructive pulmonary disease; Values with * have missing data and were imputed. Missingness: Systolic blood pressure (9.0%), Diastolic blood pressure (9.0%), BMI (37.5%), Smoking Status (19.2%).

**Supplementary Table 5. Baseline characteristics of Transformer-identified clusters (Female patients only)**

| **Cluster** | **1** | **2** | **3** | **4** | **5** | **6** | **7** |
| --- | --- | --- | --- | --- | --- | --- | --- |
| N | 2,818 | 3,087 | 2,844 | 4,308 | 5,729 | 7,010 | 7,626 |
| Mean age (in years) (SD) | 62.2 (12.1) | 80.4 (8.2) | 81.7 (8.8) | 77.4 (11.0) | 78.0 (10.5) | 82.6 (10.1) | 88.4 (6.5) |
| **Ethnicity** | | | | | | | |
| White (%) | 2,482 (88.1) | 2,850 (92.3) | 2,680 (94.2) | 3,559 (82.6) | 5,402 (94.3) | 6,566 (93.7) | 7,224 (94.7) |
| Black (%) | 105  (3.7) | 46  (1.5) | 21 (0.7) | 214  (5.0) | 60  (1.0) | 82  (1.2) | 32  (0.4) |
| Asian (%) | 66  (2.3) | 50  (1.6) | 62 (2.2) | 385  (8.9) | 127 (2.2) | 152  (2.2) | 80  (1.0) |
| Other or unknown (%) | 165  (5.9) | 141  (4.6) | 81 (2.8) | 150  (3.5) | 140 (2.4) | 210  (3.0) | 290 (3.8) |
| **Socioeconomic status quintile** | | | | | | | |
| Quintile 1 (%) | 673 (23.9) | 858 (27.8) | 723 (25.4) | 771 (17.9) | 1,136 (19.8) | 1,681 (24.0) | 2,073 (27.2) |
| Quintile 2 (%) | 572 (20.3) | 643 (20.8) | 567 (19.9) | 737 (17.1) | 1,032 (18.0) | 1,358 (19.4) | 1,633 (21.4) |
| Quintile 3 (%) | 576 (20.4) | 658 (21.3) | 578 (20.3) | 851 (19.8) | 1,164 (20.3) | 1,561 (22.3) | 1,634 (21.4) |
| Quintile 4 (%) | 484 (17.2) | 497 (16.1) | 479 (16.8) | 868 (20.1) | 1,073 (18.7) | 1,207 (17.2) | 1,187 (15.6) |
| Quintile 5 (%) | 513 (18.2) | 431 (14.0) | 497 (17.5) | 1,081 (25.1) | 1,324 (23.1) | 1,203 (17.2) | 1,099 (14.4) |
| **Smoking Status^*^** | | | | | | | |
| Smoker (%) | 768 (27.3) | 332 (10.8) | 354 (12.4) | 604 (14.0) | 1,343 (23.4) | 879 (12.5) | 786 (10.3) |
| Ex-smoker (%) | 862 (30.6) | 1,005 (32.6) | 1,028 (36.1) | 1,424 (33.1) | 2,599 (45.4) | 2,649 (37.8) | 2,523 (33.1) |
| Non-smoker (%) | 1,188 (42.2) | 1,750 (56.7) | 1,462 (51.4) | 2,280 (52.9) | 1,787 (31.2) | 3,482 (49.7) | 4,317 (56.6) |
| **Measurements** | | | | | | | |
| Mean SBP (SD) | 132.2 (18.6) | 139.0 (19.5) | 134.6 (19.2) | 136.6 (19.2) | 134.3 (18.7) | 134.6 (19.2) | 134.3 (19.5) |
| Mean DBP (SD) | 78.8 (11.3) | 76.3 (11.1) | 73.9 (10.6) | 73.3 (11.1) | 75.1 (11.1) | 74.3 (10.8) | 74.8 (10.9) |
| Mean HR (SD) | 88.5 (20.5) | 78.9 (15.9) | 73.0 (18.0) | 80.9 (19.8) | 84.3 (17.8) | 80.9 (18.4) | 79.1 (15.7) |
| Mean BMI (SD) | 28.7 (7.0) | 28.6 (6.5) | 27.2 (5.6) | 30.8 (7.0) | 27.7 (7.2) | 28.1 (6.8) | 24.8 (5.4) |
| **Risk Factors** | | | | | | | |
| Atrial fibrillation (%) | 712 (25.3) | 1,562 (50.6) | 1,117 (39.3) | 1,553 (36.0) | 2,292 (40.0) | 3,154 (45.0) | 4,017 (52.7) |
| Chronic kidney disease (%) | 458 (16.3) | 1,238 (40.1) | 1,097 (38.6) | 2,224 (51.6) | 1,972 (34.4) | 3,337 (47.6) | 3,048 (40.0) |
| COPD (%) | 145  (5.1) | 68  (2.2) | 150 (5.3) | 542 (12.6) | 3,676 (64.2) | 1,295 (18.5) | 327 (4.3) |
| Diabetes (%) | 93  (3.3) | 178  (5.8) | 234 (8.2) | 4,289 (99.6) | 727 (12.7) | 1,604 (22.9) | 387 (5.1) |
| Dyslipidaemia (%) | 294 (10.4) | 627 (20.3) | 1,202 (42.3) | 1,770 (41.1) | 1,312 (22.9) | 1,796 (25.6) | 908 (11.9) |
| Hypertension (%) | 1,023 (36.3) | 2,957 (95.8) | 2,380 (83.7) | 3,895 (90.4) | 4,201 (73.3) | 5,604 (79.9) | 5,387 (70.6) |
| Ischaemic heart disease (%) | 640 (22.7) | 705 (22.8) | 2,732 (96.1) | 2,228 (51.7) | 2,443 (42.6) | 3,064 (43.7) | 2,344 (30.7) |
| Myocardial infarction (%) | 374 (13.3) | 263  (8.5) | 1,400 (49.2) | 1,136 (26.4) | 1,159 (20.2) | 1,474 (21.0) | 1,109 (14.5) |
| Obesity (%) | 409 (14.5) | 327 (10.6) | 227 (8.0) | 1,114 (25.9) | 827 (14.4) | 853 (12.2) | 346 (4.5) |
| Stroke (%) | 176  (6.2) | 566 (18.3) | 630 (22.2) | 905 (21.0) | 950 (16.6) | 1,486 (21.2) | 1,796 (23.6) |
| Thyroid disorders (%) | 80  (2.8) | 83  (2.7) | 86 (3.0) | 220  (5.1) | 224 (3.9) | 5,240 (74.8) | 254 (3.3) |

%: percent; SD: standard deviation; BMI: body mass index; SBP: systolic blood pressure; DBP: diastolic blood pressure; HR: Heart rate; COPD: Chronic obstructive pulmonary disease; Values with * have missing data and were imputed. Missingness: Systolic blood pressure (8.2%), Diastolic blood pressure (8.2%), BMI (45.0%), Smoking Status (26.2%).

**Supplementary Table 6. Descriptive summary of Transformer-derived heart failure clusters and their characteristics.**

| **Cluster** | **Cluster Label** | **Cluster-specific Characteristics** |
| --- | --- | --- |
| 1 | Early-onset | Younger age patients, low prevalence of risk factors |
| 2 | Hypertension | High prevalence of hypertension (94.5%); high rate of prescription of antihypertensive medication, especially diuretics (85.8%) |
| 3 | IHD | High prevalence (97.0%) of IHD frequently with angina symptoms (58.7%) |
| 4 | Metabolic dysfunction | Significant presence of diabetes (99.6%), chronic kidney disease (48.7%) and hypertension (88.3%), highest mean BMI |
| 5 | COPD | High prevalence (69.6%) of COPD; high rate of prescription of bronchodilators (88.2%) and corticosteroids (49.5%) |
| 6 | Thyroid dysfunction | High prevalence of thyroid disorders (66.9%); high rate of prescription of thyroid/antithyroid medication (67.0%), mostly women (66.4%) |
| 7 | Late-onset | Older age patients and mostly women (65.0%) |

COPD: Chronic obstructive pulmonary disease; IHD: Ischemic heart disease

## Supplementary Figures


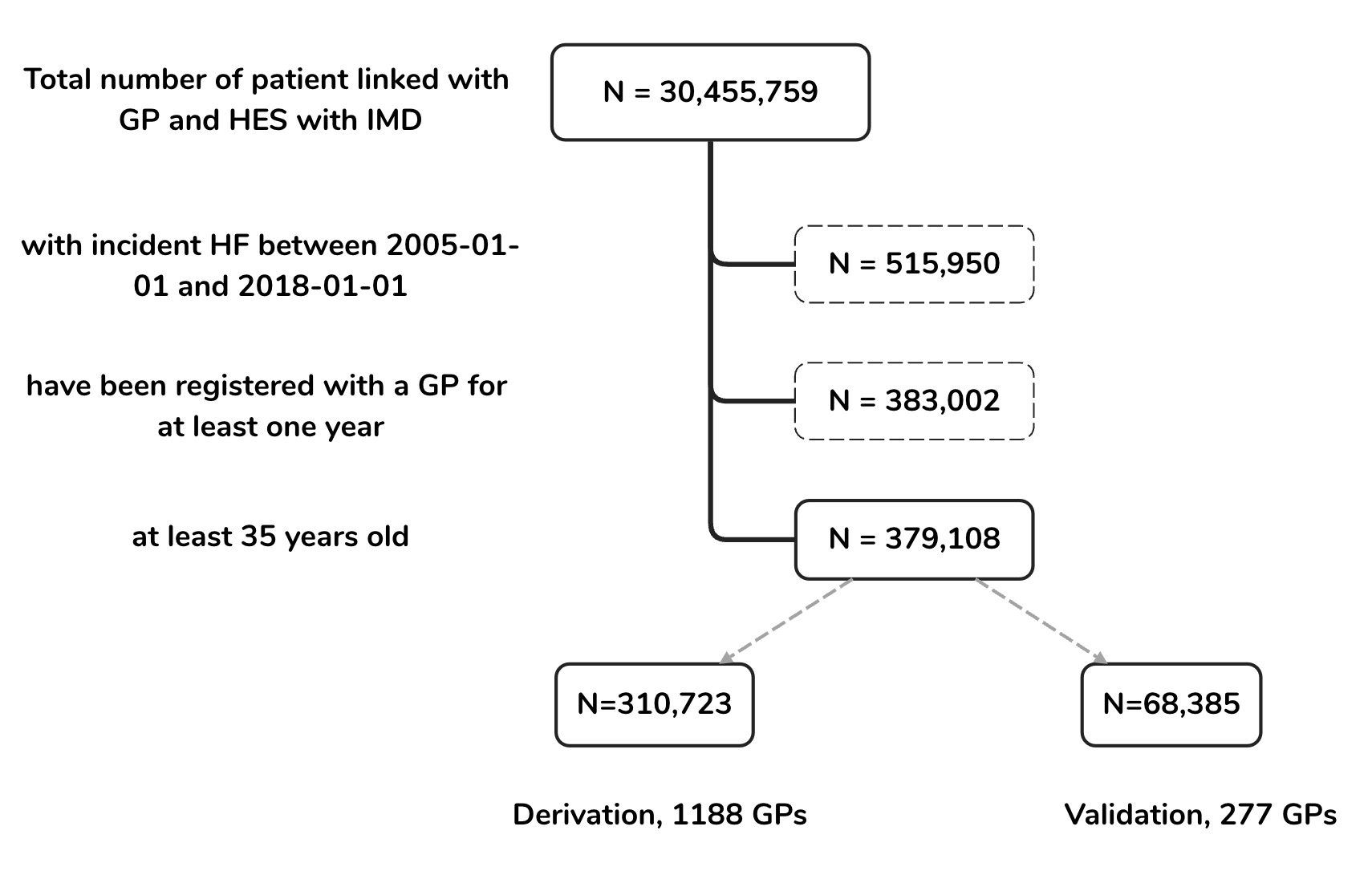


**Supplementary Figure 1. An illustration of the cohort selection pipeline.** GP: General practices; HES: Hospital episode statistics; IMD: Index of multiple deprivation

**
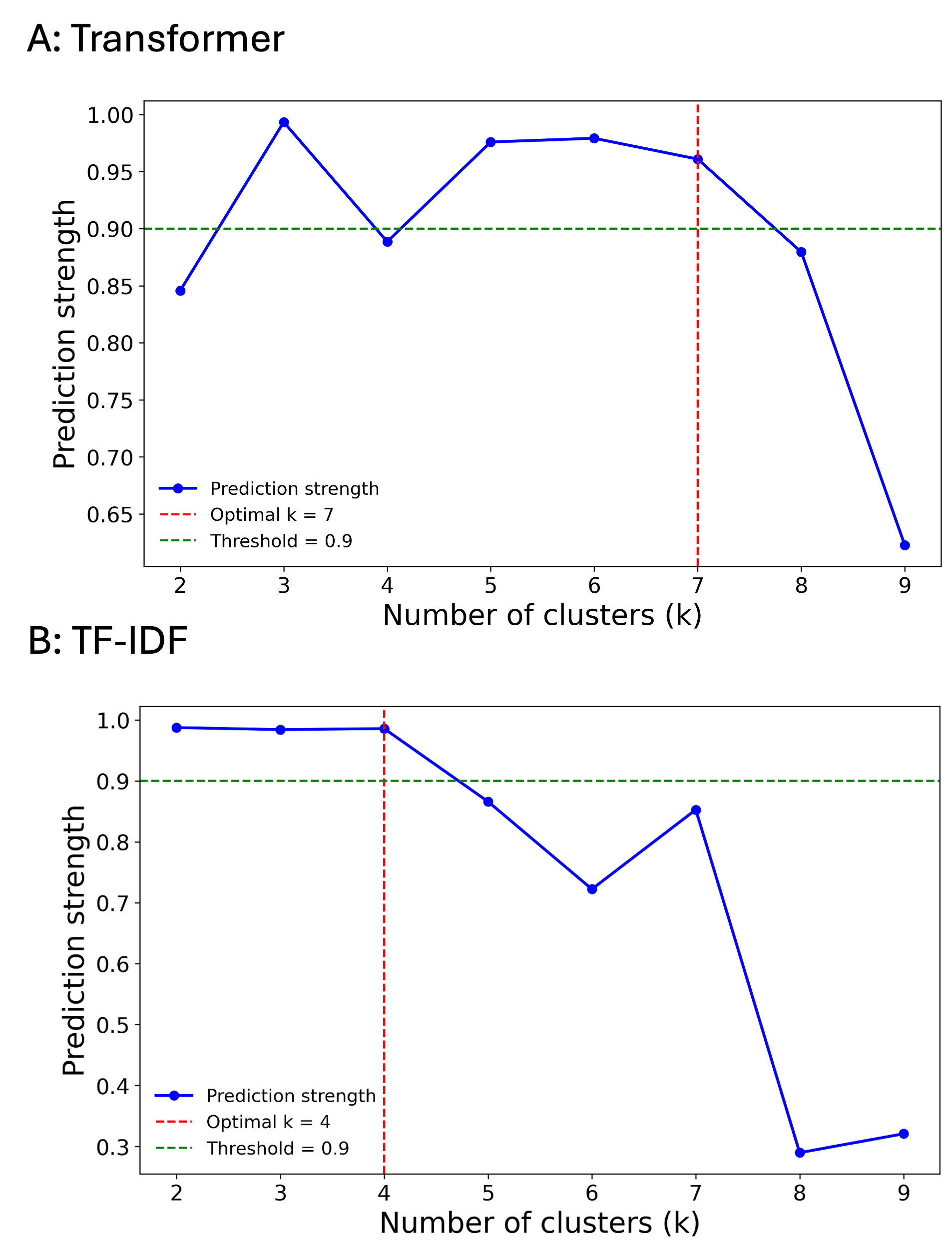
**

**Supplementary Figure 2. Prediction strength across different cluster counts.** Prediction strength measures the stability of clusters. The threshold indicates the minimum prediction strength required to identify clusters as stable. The optimal k is defined as the highest cluster count above the threshold, where the model achieves a balance between being sufficiently informative and maintaining stability. TF-IDF: Term frequency-inverse document frequency


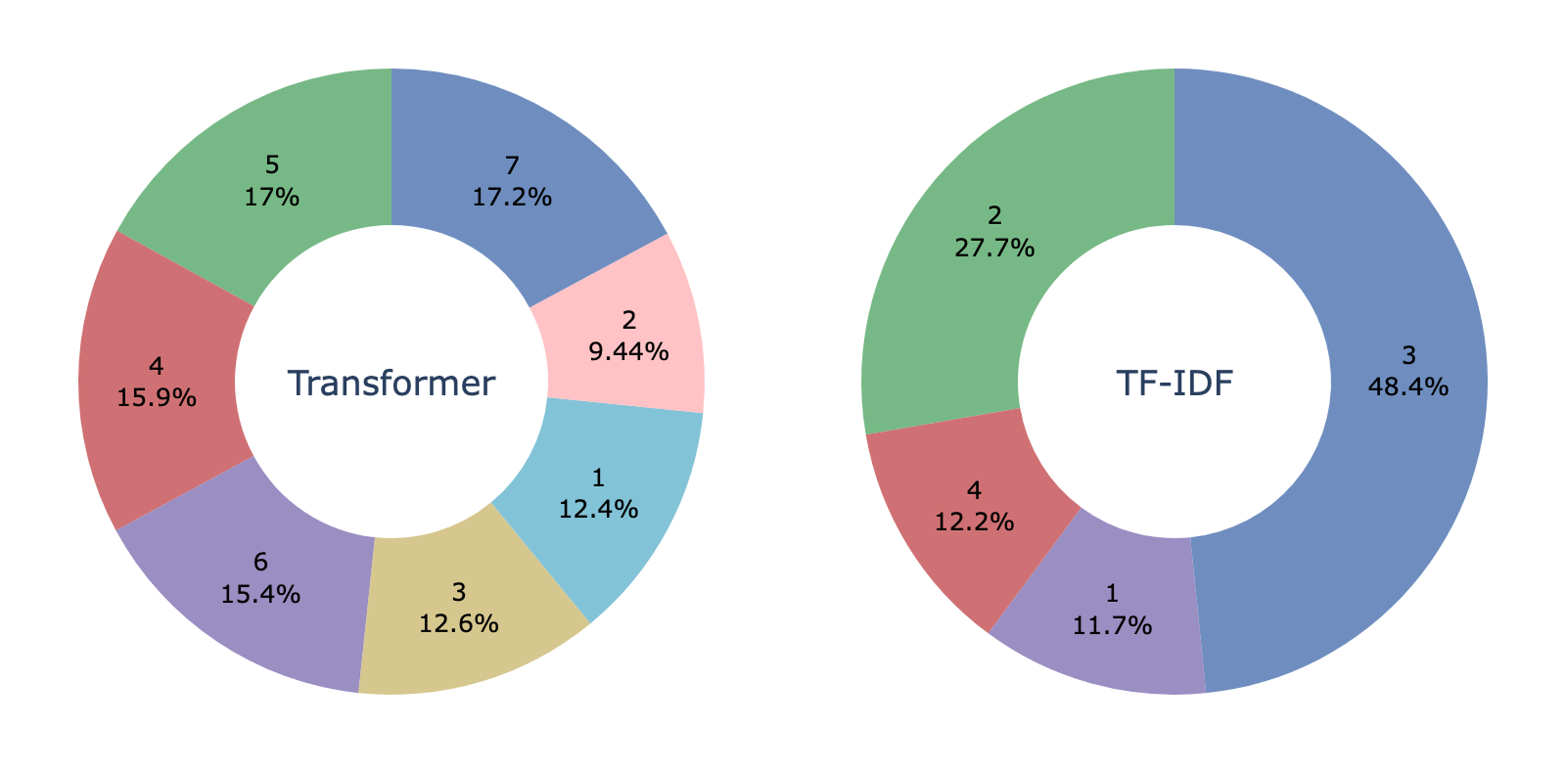


**Supplementary Figure 3. The distribution of heart failure clusters on the validation cohort identified by TF-IDF and Transformer model.** TF-IDF: Term frequency-inverse document frequency


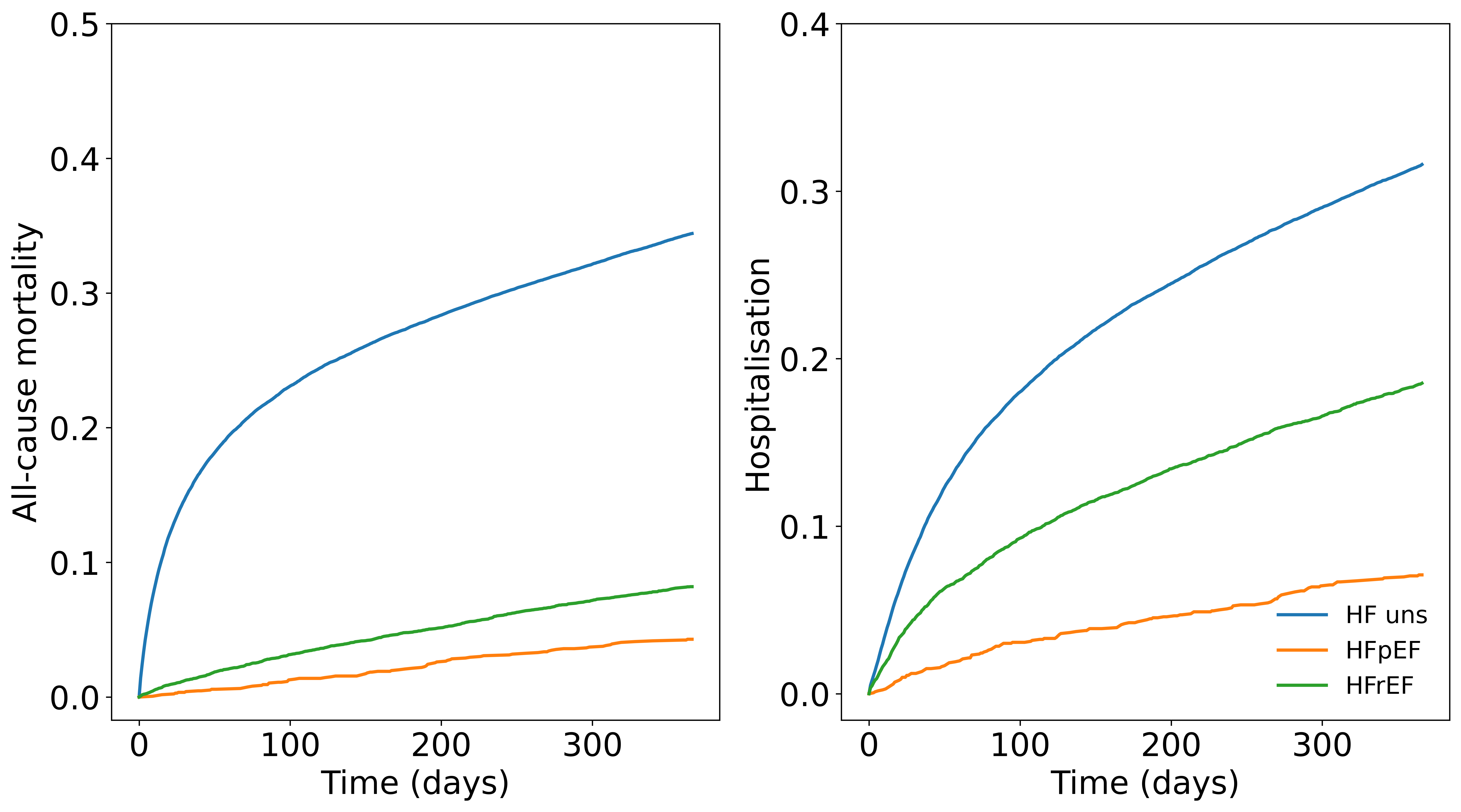


**Supplementary Figure 4**. **All-cause mortality and heart failure hospitalisation for patients with HF with reduced ejection fraction (HFrEF), HF with preserved ejection fraction (HFpEF), and unspecified HF (HF uns).**


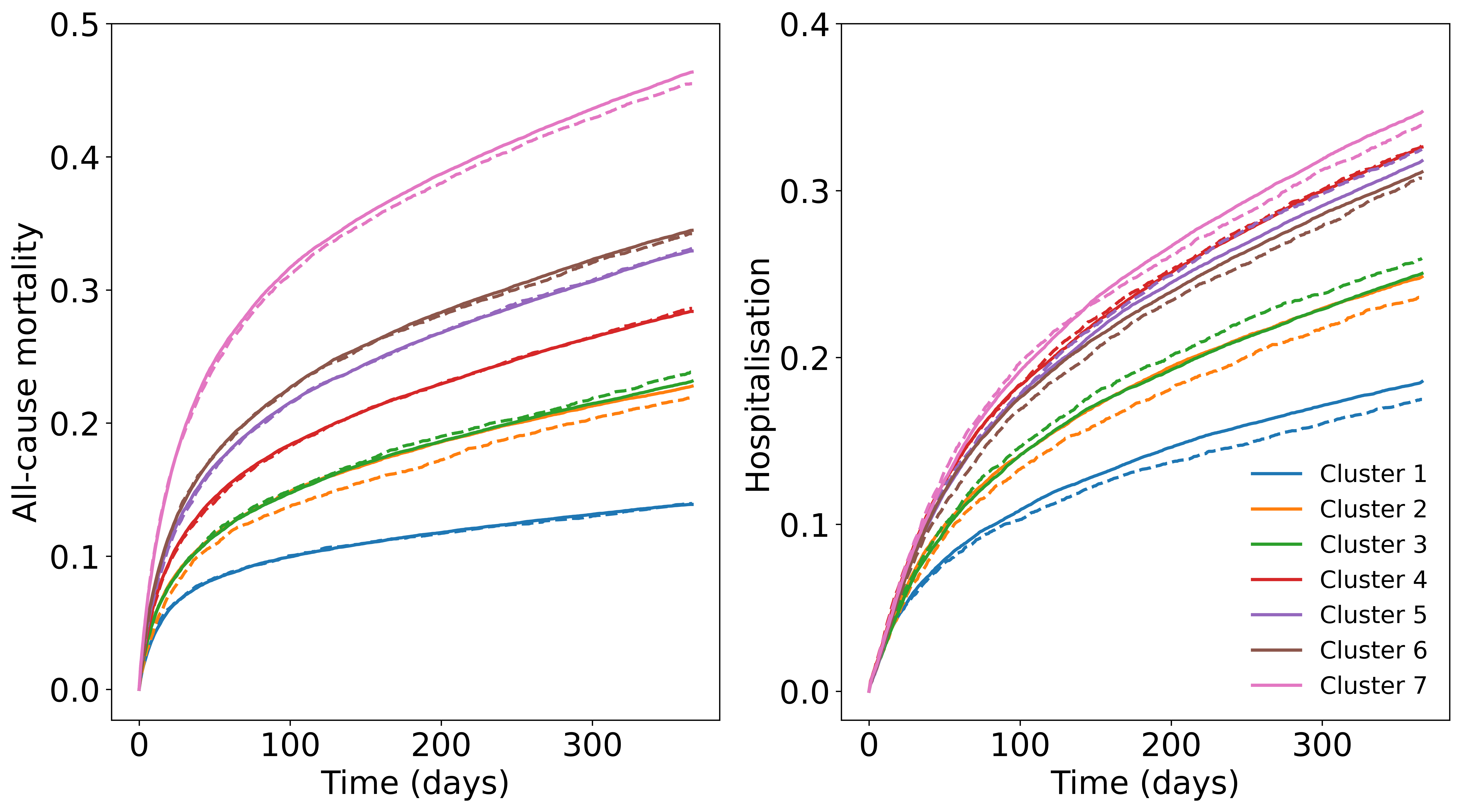


**Supplementary Figure 5**. **All-cause mortality and heart failure hospitalisation for patient clusters identified by the Transformer model on derivation and validation cohort.** The derivation cohort's survival and hospitalisation curves were juxtaposed against those from the validation cohort. Solid lines represent the derivation cohort, and dotted lines correspond to the validation cohort.


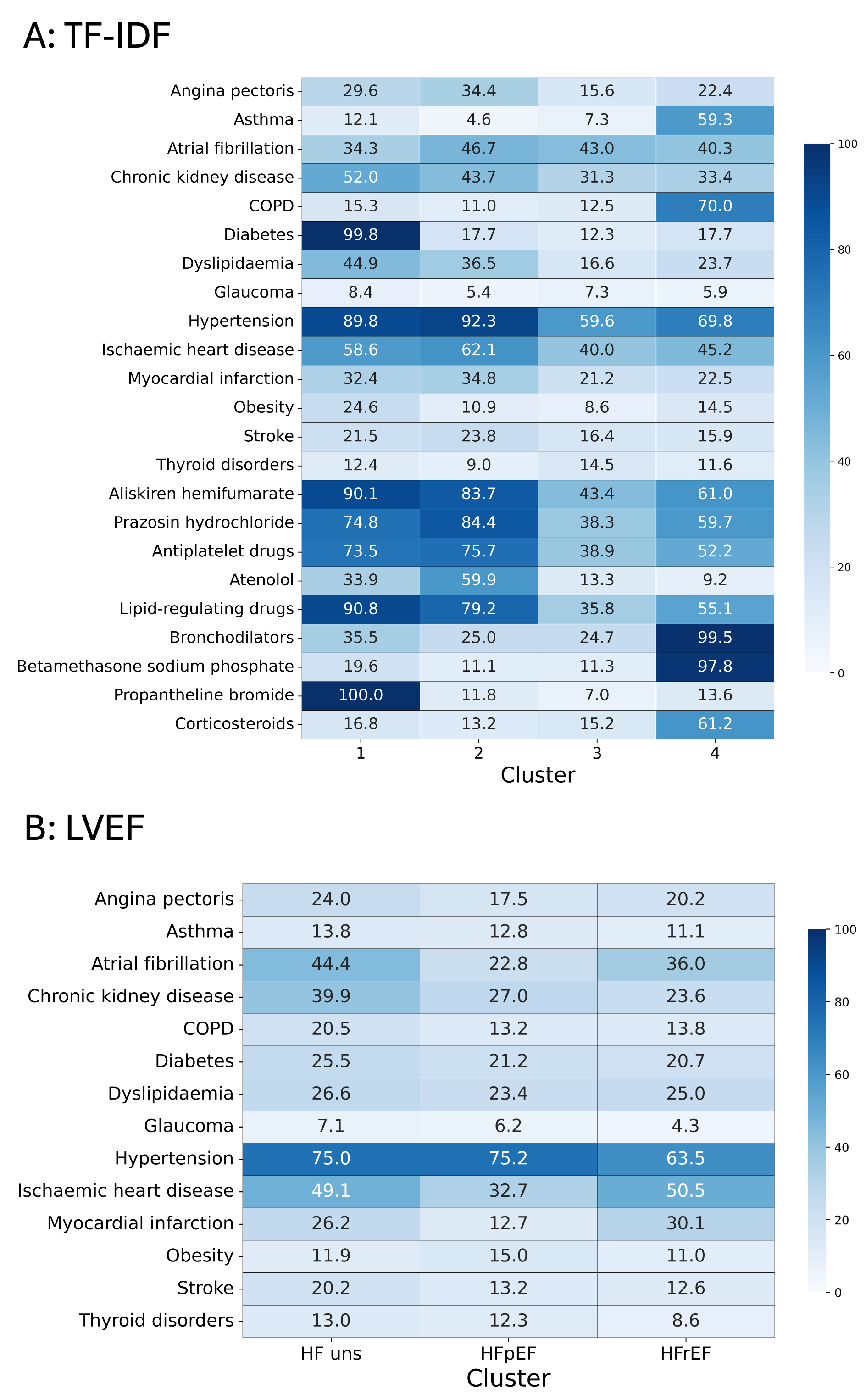


**Supplementary Figure 6. Heatmaps of TF-IDF-based and LVEF-based HF clusters showing the percentage of patients with selected factors (including comorbidities and medications) at incident HF.** Each cell indicates the proportion of patients in a cluster who had the condition. Abbreviations: TF-IDF: term frequency–inverse document frequency; LVEF: left ventricular ejection fraction; HFrEF: heart failure with reduced ejection fraction; HFpEF: heart failure with preserved ejection fraction; HF uns: unspecified HF; COPD: chronic obstructive pulmonary disease.
